# Technische Grundlagen großer Sprachmodelle

**DOI:** 10.1007/s00117-025-01427-z

**Published:** 2025-03-10

**Authors:** Christian Blüthgen

**Affiliations:** Institut für Diagnostische und Interventionelle Radiologie, Universitätsspital Zürich, Universität Zürich, Rämistrasse 100, 8091 Zürich, Schweiz

**Keywords:** Large Language Models, Generative künstliche Intelligenz, Computerlinguistik, Natürliche Sprachverarbeitung, ChatGPT, Large language models, Generative artificial intelligence, Computational linguistics, Natural language processing, ChatGPT

## Abstract

**Hintergrund:**

Große Sprachmodelle (Large Language Models, LLMs) wie ChatGPT haben die Art und Weise, wie Computer menschliche Sprache analysieren können und wie wir mit Computern interagieren können, in nur kurzer Zeit revolutioniert.

**Fragestellung:**

Überblick über die Entstehung und die Grundprinzipien von computergestützten Sprachmodellen.

**Methoden:**

Narrative literaturgestützte Beleuchtung der Entstehungsgeschichte von Sprachmodellen, der technischen Grundlagen, des Trainingsprozesses und der Limitationen großer Sprachmodelle.

**Ergebnisse:**

Große Sprachmodelle basieren heutzutage meist auf Transformer-Modellen, die durch ihren Aufmerksamkeitsmechanismus Kontext erfassen können. Durch einen mehrstufigen Trainingsprozess mit umfassendem Vortraining, überwachtem Feintuning und Alignment mit menschlichen Präferenzen haben große Sprachmodelle ein generelles Sprachverständnis entwickelt. So sind sie in der Lage, flexibel Texte zu analysieren sowie mit hoher Qualität zu erzeugen.

**Schlussfolgerung:**

Ihre technischen Grundlagen und ihr Trainingsprozess machen große Sprachmodelle zu vielseitig einsetzbaren Allzweckwerkzeugen bei der Textverarbeitung, mit zahlreichen Anwendungsmöglichkeiten in der Radiologie. Die größte Limitation ist die Tendenz, falsche, aber plausibel klingende Informationen mit hoher Konfidenz zu postulieren.

## Abstract (en)

**Background:**

Large language models (LLMs) such as ChatGPT have rapidly revolutionized the way computers can analyze human language and the way we can interact with computers.

**Objective:**

To give an overview of the emergence and basic principles of computational language models.

**Methods:**

Narrative literature-based analysis of the history of the emergence of language models, the technical foundations, the training process and the limitations of LLMs.

**Results:**

Nowadays, LLMs are mostly based on transformer models that can capture context through their attention mechanism. Through a multistage training process with comprehensive pretraining, supervised fine-tuning and alignment with human preferences, LLMs have developed a general understanding of language. This enables them to flexibly analyze texts and produce outputs of high linguistic quality.

**Conclusion:**

Their technical foundations and training process make large language models versatile general-purpose tools for text processing, with numerous applications in radiology. The main limitation is the tendency to postulate incorrect but plausible-sounding information with high confidence.

Große Sprachmodelle (Large Language Models, LLMs) wie ChatGPT haben die Art und Weise, wie Computer menschliche Sprache analysieren können und wie wir mit Computern interagieren können, in nur kurzer Zeit revolutioniert. Da die präzise und stark kontextbezogene Verwendung von Sprache ein Grundbaustein des radiologischen Alltags ist, lohnt es sich für Radiologen und andere Berufe im Bereich medizinischer Bildgebung, sich mit diesen Modellen vertraut zu machen. Um zu verstehen, wie diese Systeme medizinische Texte verarbeiten und generieren können, ist ein Blick auf ihre technischen Grundprinzipien hilfreich. Dieser Artikel bietet hierfür einen Überblick über die Entstehung und die Grundprinzipien von computergestützten Sprachmodellen.

## Große Sprachmodelle in der Landschaft maschinellen Lernens

Generative künstliche Intelligenz (KI) stellt einen bedeutenden Zweig des maschinellen Lernens (d. h. der Verbesserung einer Leistung anhand automatischen Lernens aus Trainingsdaten) dar, der sich von anderen Ansätzen wie der diskriminativen oder prädikativen Modellierung unterscheidet [1, 2]. Während diskriminative Modelle darauf ausgerichtet sind, Eingaben in vorgegebene Kategorien einzuordnen (wie beispielsweise die Klassifikation von Röntgenbildern), und prädiktive Modelle spezifische Werte vorhersagen (etwa Laborwerte), zielt die generative KI darauf ab, neue Inhalte zu erschaffen, die den Trainingsdaten nachempfunden sind, idealerweise ohne sie bloß zu kopieren. Damit sind generative Modelle in der Lage, Texte, Bilder oder auch komplexere Daten wie Videos oder dreidimensionale medizinische Bilddaten zu synthetisieren [3–5]. In den letzten Jahren haben vor allem große Sprachmodelle als eine Art von generativer KI Aufmerksamkeit durch ihre Fähigkeit erregt, auf hohem Niveau in Form von menschlicher Sprache mit Anwendern zu kommunizieren.

Allgemein gesprochen ist ein Sprachmodell ein mathematisches Modell, das eine sequenzielle Abfolge von Elementen (z. B. Worte eines Satzes) modelliert. Ein generatives Sprachmodell sagt dabei in Abhängigkeit eines Eingabetextes eine Wahrscheinlichkeitsverteilung für die als Nächstes in der Sequenz auftauchenden Textelemente voraus. Computergestützte Sprachmodelle sind keine neue Erfindung. Ihre Entwicklung verlief über mehrere Epochen: von einfachen statistischen Modellen der 1990er-Jahre, die z. B. auf Worthäufigkeiten oder Häufigkeiten von Wortkombinationen basierten und für spezifische Aufgaben konstruiert wurden, über erste neuronale Architekturen ab 2013 (die mittels mehrschichtiger Signalverarbeitungseinheiten über verschachtelte, hierarchische Abstraktionen flexibel Muster erkennen können), die für flexiblere Aufgaben wie Textklassifikation benutzt werden konnten, bis hin zu den heutigen großen Sprachmodellen. Im Unterschied zu früheren Modellen sind heutige LLMs auf gewaltigen Textmengen vortrainiert und können anschließend für zahlreiche Aufgaben ohne weiteres Training flexibel benutzt oder, falls nötig, spezifisch feingetunt (d. h. präzise angepasst) werden [6].

**Abb. 1:**
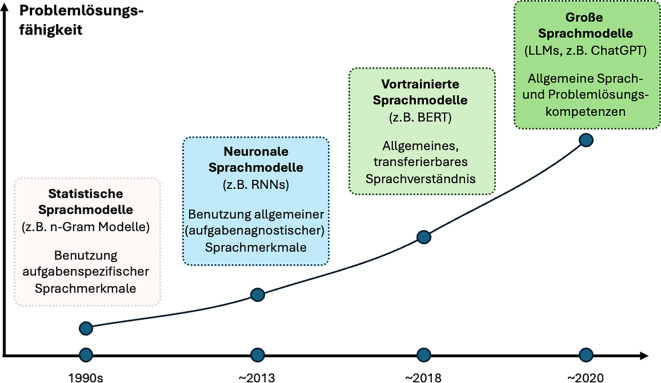
Vereinfachte Darstellung der Entwicklung computergestützter Sprachmodelle hinsichtlich ihrer Problemlösungsfähigkeit im Verlauf der Zeit. Die Jahresangaben sind hier symbolisch gewählt, da die Entstehung von großen Sprachmodellen mit wenigen Ausnahmen nicht sprunghaft verläuft, sondern ein Prozess mit vielen einzelnen inkrementellen Entwicklungen ist.* BERT* Bidirectional Encoder Representations from Transformers, *GPT* Generative pretrained transformer, *LLM* Large Language Model, *n‑Gramm* Ergebnis einer Zerlegung von Text in kleinere Fragmente, *RNN* Rekurrentes neuronales Netzwerk. (Adaptiert nach [7]).

Die neueste Generation der Sprachmodelle, zu der bekannte Systeme wie ChatGPT (OpenAI, USA) und Claude (Anthropic, USA) gehören, zeichnet sich durch eine bemerkenswerte Flexibilität und Universalität in der Aufgabenbewältigung aus [8]. Diese Modelle können nicht nur typische Aufgaben der Sprachverarbeitung bewältigen, sondern zeigen mitunter auch Fähigkeiten im logischen Schlussfolgern, Problemlösen und sogar im Verstehen impliziter Kontexte. Sie haben sich von reinen Textverarbeitungswerkzeugen mit engem Anwendungsbereich zu immer vielfältigeren Problemlösern entwickelt, wie die obige Abbildung (Abb. 1) verdeutlicht.

## Technische Grundlagen moderner Sprachmodelle

### Von Text zu Zahlen: Tokenisierung und Einbettung

**Abb. 2:**
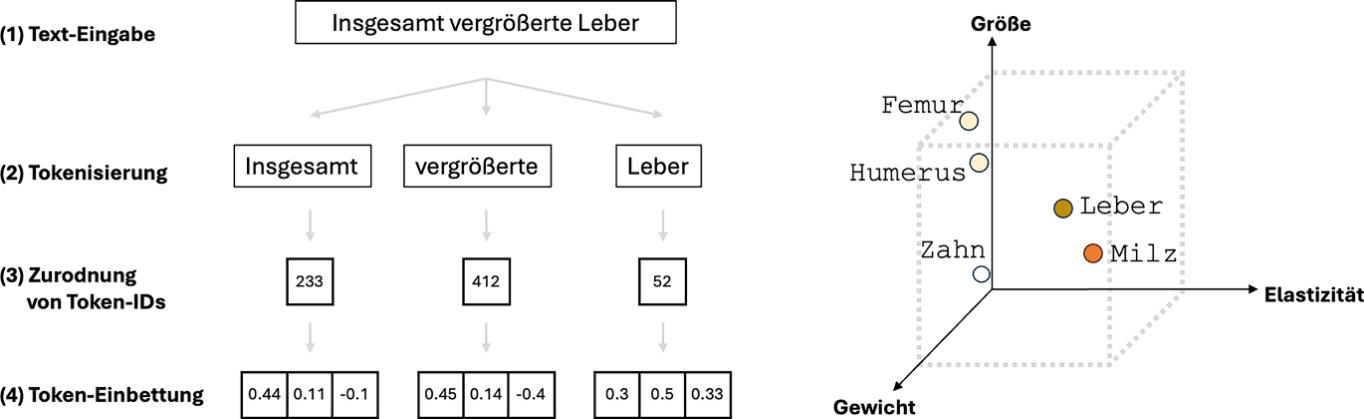
Schematische Übersicht über Tokenisierung und Einbettung. *Links* Vorgang der Token-Einbettung am Beispiel des Satzes „Insgesamt vergrößerte Leber“. Der Eingabesatz *(1) *wird zunächst in kleinere Bestandteile (Token) zerlegt *(2)*. Diese Token werden mithilfe eines festgelegten Wörterbuchs in Token-IDs (im Wörterbuch gespeicherte Indizes für eine bestimmte Zeichenkette) umgewandelt *(3)* und anschließend als mehrdimensionaler Vektor (hier: drei Dimensionen) repräsentiert *(4)*. *Rechts* Beispiel eines fiktiven dreidimensionalen Vektorraums, bestehend aus den Dimensionen Größe, Elastizität und Gewicht. Anatomische Strukturen können in diesem Raum abgebildet werden. Strukturen mit ähnlichen Eigenschaften (z. B. parenchymale Oberbauchorgane) kommen in diesem Raum nah beieinander zu liegen. Die Dimensionen bei der Einbettung von Text in LLMs sind zahlreicher, abstrakter und automatisch erlernt, anstatt wie in diesem Beispiel vorher festgelegt

Der erste Schritt in der maschinellen Textverarbeitung durch LLMs ist die Tokenisierung (Abb. 2; [9]). Dabei wird der Eingabetext in kleinere Einheiten – sog. Token – zerlegt. Token können einzelne Wörter, Wortteile oder auch Satzzeichen sein. Ein Satz wie „Kein Hinweis auf Pneumothorax“ wird beispielsweise in Token wie „Kein“, „Hinweis“, „auf“ und „Pneumothorax“ zerlegt. Da neuronale Netze nur mit Zahlen arbeiten können, wird anschließend jedes Token durch einen hochdimensionalen Zahlenvektor repräsentiert – die sog. Einbettung („embedding“). Diese Vektoren sind so konstruiert, dass die Vektoren von Token mit ähnlicher Bedeutung auch ähnliche Zahlenwerte und damit eine gewisse Nähe zueinander in einem erlernten, hochdimensionalen mathematischen Raum aufweisen. Die Einbettungen sind bei den meisten Modellen erlernt und werden in einer Art Wörterbuch (Vokabular) unter einer für jeden Token einzigartigen Nummer (Token-ID) abgespeichert.

### Die Transformer-Architektur: das Herzstück moderner Sprachmodelle

Das zentrale Element der meisten modernen Sprachmodelle ist die Transformer-Architektur [10]. Diese besteht aus zwei Hauptkomponenten: einem *Encoder*, der den Eingabetext verarbeitet, und einem *Decoder*, der den Ausgabetext generiert. Heutige LLMs wie GPT nutzen jedoch oft ausschließlich den Decoder-Teil, da sich dieser als besonders effektiv für die Textgenerierung erwiesen hat. Die Eingabesequenz muss hierfür (wie bei der klassischen Encoder-Decoder-Variante) tokenisiert und in eine Token-Einbettung überführt werden.

**Abb. 3:**
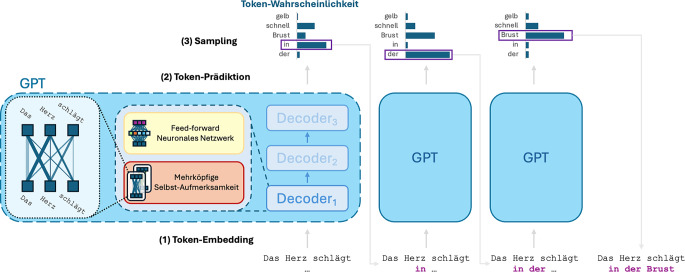
Vereinfachte Übersicht über die Vorhersage der nächsten drei Worte in der Eingabesequenz „Das Herz schlägt“ mittels eines Generative-Pretrained-Transformer(*GPT*)-Modells. (*1*) Die Eingabesequenz wird in mehrere Bestandteile (Token) zerlegt (Tokenisierung) und in eine Vektorrepräsentation überführt (Einbettung oder Embedding). (*2*) Dieser Vektor wird von in Serie geschalteten, identischen Decoder-Blöcken prozessiert. Dabei erzeugen in jedem Block mehrköpfige Selbst-Aufmerksamkeitsmodule („multi-headed self-attention“) weiter abstrahierte, durch den Attention-Mechanismus kontextbewusste Vektorrepräsentationen. In nachgeschalteten Feed-Forward-neuronalen-Netzwerken werden diese Repräsentationen weiter prozessiert, was die Fähigkeit des Transformers für komplexere Zusammenhänge erhöht. Dieser Schritt wird mehrfach durchlaufen. Am Ende wird eine Wahrscheinlichkeitsverteilung für den nächsten Token vorhergesagt, aus der durch (*3*) Sampling der wahrscheinlichste nächste Token ausgewählt wird („in“, markiert durch eine violette Box). Dieser Prozess wiederholt sich für die Vorhersage der folgenden Token („der“ und „Brust“), wobei die jeweils zuvor vorhergesagten Token mit in den Kontext einfließen. (*GPT* Generative Pretrained Transformer)

Der Decoder (Abb. 3) besteht aus mehreren in Serie geschalteten identischen Blöcken (sog. Transformer-Blöcken), die jeweils zwei Hauptkomponenten enthalten: den Attention-Mechanismus und ein Feed-Forward-Netzwerk. Der Attention-Mechanismus ermöglicht es dem Modell, die Beziehungen zwischen allen Wörtern eines Textes zu analysieren. Dabei wird für jedes Wort ermittelt, welche anderen Wörter im aktuellen Kontext besonders relevant sind. In dem Satz „Die Infiltrate im rechten Lungenoberlappen haben sich zurückgebildet“ sollte der Attention-Mechanismus beispielsweise automatisch berechnen, dass sich das Wort „sich“ auf „die Infiltrate“ bezieht. Diese Fähigkeit, Kontext mit einzubeziehen, ist essenziell zur korrekten Erfassung medizinischer Zusammenhänge: So kann das Wort „Bruch“ sich auf Knochen, aber auch auf Fremdmaterial wie eine Sternalzerklage beziehen. Da der Attention-Mechanismus konstruktionsbedingt selbst keine Möglichkeit hat, die Reihenfolge der Wörter in der Eingabe zu berücksichtigen, wird normalerweise zusätzlich eine Positionskodierung zu den Embeddings addiert, mit der die Position der Wörter im Text abgebildet werden kann [11].

Das Feed-Forward-Netzwerk ist ein einfaches neuronales Netzwerk (ein mehrschichtiges Perzeptron), welches dazu dient, die durch den Attention-Mechanismus gewonnenen Informationen weiter zu verarbeiten, und somit ermöglicht, komplexere Muster zu erkennen. Die Kombination aus Attention-Mechanismus und Feed-Forward-Netzwerk befähigt das Modell, sowohl lokale als auch globale Zusammenhänge im Text zu erfassen.

Eine wichtige technische Einschränkung der Transformer-Architektur ist das Kontextfenster. Es definiert, wie viele Token das Modell gleichzeitig verarbeiten und „im Arbeitsgedächtnis“ behalten kann. Bei GPT‑3.5 beträgt diese Grenze beispielsweise etwas über 4000 Token, bei neueren Modellen auch deutlich mehr. Texte, die diese Länge überschreiten, müssen in kleinere Abschnitte aufgeteilt werden, wobei das Modell die Informationen außerhalb des aktuellen Kontextfensters nicht direkt berücksichtigen kann. Für medizinische Anwendungen ist diese Begrenzung z. B. bei der Verarbeitung längerer Krankenakten oder der Analyse mehrerer zusammenhängender Befunde relevant. Ein LLM kann beispielsweise nicht die gesamte Krankengeschichte eines Patienten auf einmal analysieren, wenn diese die Länge des Kontextfensters überschreitet. Das Kontextfenster ist zudem relevant für alle LLM-bezogenen Methoden, die auf eine Anreicherung des Kontexts mit potenziell nützlichen Informationen abzielen, beispielsweise bei der Retrieval-augmented Generation (RAG, der automatisierten Miteinbeziehung von möglicherweise für die Ausgabe relevanten Informationen aus Datenbanken oder anderen Quellen) oder beim sog. In-context Learning (der Bereitstellung von Beispielen von Eingaben und wünschenswerten Antworten).

### Textgenerierung und Sampling-Strategien

**Abb. 4:**
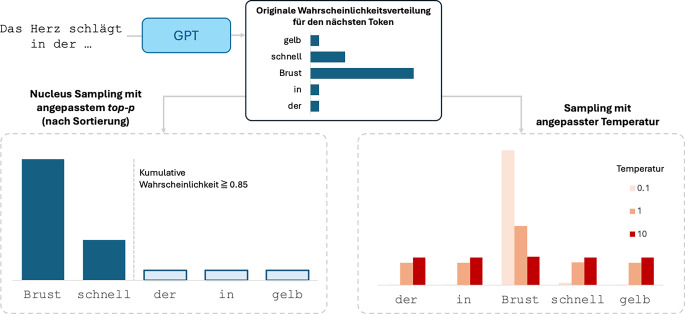
Sampling-Strategien zur Textgenerierung in Sprachmodellen. Für die Eingabesequenz „Das Herz schlägt in der …“ berechnet das GPT-Modell zunächst eine Wahrscheinlichkeitsverteilung über mögliche nachfolgende Tokens (*oben*). Diese Verteilung kann durch verschiedene Sampling-Methoden modifiziert werden: Beim Nucleus-Sampling (*unten links*) werden nur die wahrscheinlichsten Token berücksichtigt, deren kumulierte Wahrscheinlichkeit einen Schwellenwert (hier top-*p* = 0,85) nicht überschreitet. Unwahrscheinliche Token außerhalb dieses Bereichs (*nicht ausgefüllte Balken*) werden beim Sampling nicht mehr berücksichtigt. Das temperaturbasierte Sampling (*unten rechts*) skaliert die gesamte Verteilung durch einen Temperaturparameter, wobei niedrige Temperaturen (T = 0,1) die Präferenz für wahrscheinliche Token verstärken und hohe Temperaturen (T = 10) zu einer gleichmäßigeren Verteilung führen (d. h. auch unwahrscheinlichere Token können mit einer ähnlichen hohen Wahrscheinlichkeit gezogen werden). *GPT* Generative Pretrained Transformer

Bei der Textgenerierung berechnet das Modell schrittweise für jede neue Position in der Sequenz eine Wahrscheinlichkeitsverteilung über alle möglichen nächsten Token im Vokabular, in Abhängigkeit des aktuellen Kontextfensters (d. h. unter Berücksichtigung der initialen Eingabe und aller bislang generierten Token, auch *autoregressive *Generation genannt). Die Auswahl des nächsten Tokens erfolgt jeweils durch Ziehen (*Sampling*) aus dieser Wahrscheinlichkeitsverteilung – ein Prozess, der durch verschiedene Parameter gesteuert werden kann (Abb. 4).

Die „Temperatur“ hat als Sampling-Parameter Einfluss auf die Form der Wahrscheinlichkeitsverteilung [12]. Bei einer niedrigen Temperatur (nahe 0) wählt das Modell bevorzugt Wörter mit hoher Wahrscheinlichkeit, was zu konsistenteren, aber möglicherweise weniger kreativen Texten führt. Eine höhere Temperatur (> 1) führt zu vielfältigeren, aber potenziell weniger fokussierten Ausgaben.

Ein weiterer wichtiger Parameter ist „Top-p“ [13]. In dem auch als Nucleus-Sampling bezeichneten Verfahren (da nur der *Kern* der Verteilung für die Ziehung betrachtet wird) begrenzt dieser Parameter die Auswahl auf die wahrscheinlichsten Wörter, deren kumulative Wahrscheinlichkeit einen bestimmten Schwellenwert (top-p) nicht überschreitet.

Da es bei medizinischer Kommunikation weniger auf Kreativität ankommt, ist eine geeignete Wahl dieser Parameter wichtig. Für sachliche Texte wie Befundberichte sollten tendenziell niedrige Werte für Temperatur und Top‑p gewählt werden, um konsistente und präzise Formulierungen zu gewährleisten. Bei der allgemeinen Texterstellung z. B. für die Patientenkommunikation hingegen kann eine etwas höhere Temperatur sinnvoll sein, um natürlicher klingende Texte zu erzeugen.

Die sequenzielle Art der Textgenerierung der Sprachmodelle macht sie anfällig für sog. Konfabulationen (in der LLM-Literatur oft – medizinisch unpräzise [14] – als Halluzinationen bezeichnet) – das Erzeugen plausibel klingender, aber faktisch falscher Informationen. Da jedes neu generierte Token Teil des Kontexts für alle nachfolgenden Entscheidungen wird, können sich ungenaue oder falsche Auswahlen verstärken. Ein Beispiel aus der Radiologie verdeutlicht diesen *Schneeballeffekt*: Wenn das LLM bei einem Thorax-CT-Befund zunächst korrekt „Im rechten Unterlappen“ generiert, könnte es bei der Beschreibung der Pathologie mit „zeigt sich“ fortfahren. Diese neutrale, aber noch unvollständige Formulierung erhöht dann die Wahrscheinlichkeit für weitere spezifizierende, aber möglicherweise erfundene Details wie „eine Raumforderung“ oder konkrete Größenangaben („3,5 cm messend“) – Informationen, die im ursprünglichen Bildmaterial gar nicht zu sehen waren. Diese Tendenz zur Detailgenerierung wird noch verstärkt, da das Modell darauf trainiert ist, vollständige und plausibel klingende medizinische Befunde zu erzeugen. Anwender von großen Sprachmodellen sind daher dringend angehalten, die faktische Richtigkeit der generierten Ausgaben zu überprüfen.

## Training und Entwicklung großer Sprachmodelle

**Abb. 5:**
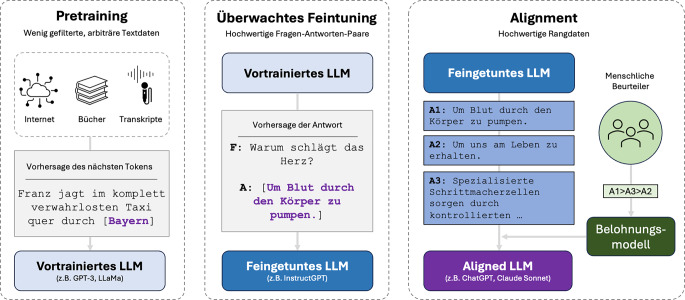
Die dreistufige Entwicklung moderner großer Sprachmodelle. Das initiale Pretraining (*links*) erfolgt auf umfangreichen, wenig gefilterten Textdaten aus diversen Quellen (z. B. Internet, Bücher und Audio-Transkripte), wobei das Modell lernt, das jeweils nächste Token in einer Sequenz vorherzusagen. In der zweiten Phase des überwachten Feintunings (*Mitte*) wird das vortrainierte Modell mit hochwertigen Frage-Antwort-Paaren weiter trainiert, um die Qualität und Nützlichkeit der Antworten zu verbessern. Die finale Alignment-Phase (*rechts*) nutzt menschliche Bewerter, um verschiedene Modellantworten (A1, A2, A3) zu rangieren. In diesem Beispiel präferierten die Beurteiler die direkte Antwort (A1) gegenüber der weniger spezifischen (A2) und der korrekten, aber sehr ausführlichen Antwort (A3). Diese Bewertungen dienen als Trainingssignal für ein Belohnungsmodell, das wiederum das LLM durch Verstärkungslernen so optimiert, dass es besser mit menschlichen Werten und Präferenzen übereinstimmt. *GPT* Generative Pretrained Transformer, *LLaMA* Large Language Model Meta AI, *LLM* Large Language Model (großes Sprachmodell)

Die Entwicklung eines LLMs ist ein mehrstufiger Prozess, der verschiedene Trainingsmethoden kombiniert (Abb. 5; [7]). Das Nachvollziehen der Trainingsprozesse ermöglicht das Verständnis mancher der einzigartigen Eigenschaften dieser Modelle. Dieser Abschnitt beschreibt die wichtigsten Phasen der LLM-Entwicklung.

### Pretraining: die Grundausbildung

Das Vortraining (*Pretraining*) bildet das Fundament eines jeden LLMs. In dieser Phase lernt das Modell grundlegende sprachliche Fähigkeiten durch die Analyse enormer Textmengen aus dem Internet, digitalen Büchern und anderen Quellen. Das Ziel ist es, grundlegende Fähigkeiten mit allgemeiner Kompetenz für Sprache, Fakten und semantische Zusammenhänge aufzubauen.

Der Trainingsprozess ist ein Beispiel für selbstüberwachtes Lernen („self-supervised learning“): Das Modell versucht, teilweise maskierte (d. h. mit Lücken versehene) oder unvollständige Texte zu vervollständigen. Dabei muss es Wörter oder Textpassagen vorhersagen, die im Original vorhanden sind, aber für das Training ausgeblendet wurden. Durch diese Aufgabe entwickelt das Modell eine grundlegende Kompetenz für Grammatik, Syntax sowie semantische Zusammenhänge zwischen Wörtern und Konzepten. Es reichert außerdem Faktenwissen aus den Trainingsdaten an, welches jedoch nur begrenzt verlässlich ist. Zum einen wird die riesige Menge an Informationen aus diversen, oft nicht validierten Quellen („Masse statt Klasse“) für das Training herangezogen. Zum anderen unterliegt das Erfassen komplexer Informationen in diesem Schritt verlustreicher Kompression (die Modelle benötigen weniger Speicherplatz als die Daten, anhand derer sie trainiert werden).

Das Pretraining ist äußerst rechenintensiv und kann bei großen Modellen selbst mit Unterstützung von Tausenden von High-End-Grafikkarten mehrere Wochen dauern. Die benötigte Hardware und Infrastruktur sowie die technische Expertise sind entsprechend teuer, mit wenig Spielraum für wiederholte Experimente oder Fehlschläge. Dies erklärt auch, warum die Entwicklung fortgeschrittener LLMs bisher hauptsächlich von großen Technologieunternehmen oder spezialisierten KI-Forschungslaboren vorangetrieben wird.

Mittlerweile sind die meisten größeren LLMs wie ChatGPT, Claude Sonnet oder Llama 3 (Meta AI, USA) aufgrund der reichen Auswahl an Pretraining-Datensätzen vielsprachig, unterstützen also auch deutschsprachige Ein- und Ausgaben.

### Überwachtes Feintuning: zielgerichtetes Training

Auf das Pretraining folgt das überwachte Feintuning („supervised fine-tuning“, SFT). Während das Pretraining dem Modell allgemeine sprachliche Fähigkeiten vermittelt, zielt das SFT darauf ab, das Modell für spezifische Anwendungsfälle zu optimieren.

Während das Trainingsziel des Pretrainings beibehalten wird (die Vorhersage des jeweils nächsten Token), liegt der Unterschied in den Trainingsdaten: Statt möglichst großer Mengen allgemeiner Texte werden nun sorgfältig kuratierte Beispieldialoge verwendet. Diese bestehen aus Paaren von Eingabetext (Prompt) und gewünschter Ausgabe (Response). Durch das Training mit diesen Beispielen lernt das Modell, welche Tokensequenzen in bestimmten Anwendungskontexten am wahrscheinlichsten und angemessensten sind.

Eine spezielle Form des SFT ist das „Instruction Tuning“ [15], bei dem das Modell durch entsprechende Trainingsdaten lernt, sprachliche Anweisungen präzise umzusetzen. Diese Fähigkeit ist essenziell für die praktische Anwendung (und damit den Erfolg aktueller LLMs wie ChatGPT), da Benutzer ihre Anfragen in Form von Instruktionen in natürlicher Sprache formulieren können.

Auch hinsichtlich einer Spezialisierung für radiologische Belange kann SFT eine Rolle spielen. In einem radiologischen Kontext könnten solche Eingabe-Ausgabe-Paare zum Beispiel beschreibende Befunde und daraus abgeleitete Beurteilungen sein, mit der Anweisung, den Befund in eine Beurteilung umzuwandeln. Dabei müssen die Trainingsdaten hohen Qualitätsstandards genügen und von Experten erstellt oder kuratiert werden, um die erforderliche Präzision und fachliche Genauigkeit sicherzustellen.

### Alignment: Abstimmung mit menschlichen Präferenzen

Nach Pretraining und Feintuning hat das LLM allgemeine Sprachkompetenzen erworben und kann Aufgaben lösen, allerdings können die Antworten in diesem Stadium oft noch in einer unerwünschten Form erfolgen, die nicht durchschnittlichen Anwenderpräferenzen entspricht. Der dritte wichtige Schritt ist daher das Alignment – die Abstimmung des Modellverhaltens mit menschlichen Werten und Präferenzen. Hierfür wurden verschiedene Methoden erfunden, wobei Reinforcement Learning from Human Feedback (RLHF) derzeit am häufigsten zum Einsatz kommt [16].

Bei RLHF bewerten menschliche Beurteiler zunächst mehrfach gewonnene Modellantworten für die gleiche Eingabe (da das Sprachmodell aus einer Verteilung zieht, sind die Antworten idealerweise unterschiedlich und lassen die Einordnung auf einer Rangliste zu). Aus diesen Bewertungen wird ein Belohnungsmodell („reward model“) trainiert, das lernt vorherzusagen, welche Antworten Menschen als besonders hilfreich oder hochwertig einstufen würden. Anschließend wird das LLM mittels bestärkenden Lernens („reinforcement learning“ – der Prozess, anhand positiven oder negativen Feedbacks zu lernen) optimiert, um die vom Belohnungsmodell vorhergesagten Bewertungen zu maximieren. Dieser Prozess führt zu Modellen, die hilfreichere und präzisere Antworten generieren und dabei eher ethische Richtlinien einhalten sowie einen konsistenten und tendenziell angemesseneren Kommunikationsstil entwickeln. Ein Anwendungsbeispiel in der Radiologie wäre das Bestreben, dem Sprachmodell einen bestimmten, im Haus bevorzugten Befundstil bei dem Entwerfen radiologischer Texte beizubringen.

## Zugang zu großen Sprachmodellen

Für den Einsatz von großen Sprachmodellen existieren verschiedene Zugangswege, die sich in Hinblick auf Flexibilität, Datenschutz und technische Anforderungen unterscheiden und anwendungsspezifische Abwägungen erfordern [17]. Die einfachste Variante stellen Web-Interfaces (wie bei ChatGPT oder Claude) dar. Diese sind sofort nutzbar, erfordern keine technische Implementation oder Vorkenntnisse, werden kontinuierlich aktualisiert, bieten jedoch wenig Anpassungsmöglichkeiten und setzen eine permanente Internetverbindung voraus.

Flexibler ist die Nutzung über APIs (Application Programming Interfaces). Hierbei können die Modelle in eigene Anwendungen integriert werden, wobei die Verarbeitung weiterhin auf den Servern der Anbieter erfolgt. Die API-Nutzung ermöglicht eine präzise Steuerung der Modellhyperparameter und die Integration in bestehende Arbeitsabläufe, etwa durch die automatische Verarbeitung von Befunden.

Für Institutionen mit hohen Datenschutzanforderungen oder speziellen Anpassungswünschen besteht die Möglichkeit, Sprachmodelle auf eigener Hardware zu betreiben, was vollständige Kontrolle über Datenflüsse und Modellkonfiguration ermöglicht. Dies erfordert jedoch für größere Modelle erhebliche Rechenressourcen und je nach Anwendungsfall substanzielles technisches Verständnis. Eine Alternative kann das Verwenden von durch sog. parametereffiziente Verfahren (z. B. QLoRA, „quantized low-rank adaptation“) hinsichtlich Speicher- und Rechenaufwand optimierter Modelle sein – mit je nach Szenario in Kauf zu nehmenden Einbußen bei der Leistungsfähigkeit der Modelle.

## Fazit für die Praxis

- Große Sprachmodelle (LLMs) sind eine neue Klasse mathematischer Modelle, die mittels extensivem Rechenaufwand auf massiven Datensätzen erstellt wurden und eine allgemeine Sprachkompetenz entwickelt haben, die das Bewerkstelligen einer Vielzahl sprachbezogener Aufgaben mit relativ geringem Aufwand ermöglicht.
- Die trainings- und designbedingte generelle Anfälligkeit von LLMs für das Erzeugen plausibel klingender, aber falscher oder potenziell schädlicher Ausgaben erfordert ein grundlegendes Verständnis ihrer Funktionsweise und menschliche Aufsicht für den verantwortungsvollen Einsatz.
- Für die Radiologie und viele andere medizinische Disziplinen bieten LLMs unter diesen Voraussetzungen eine neue, mächtige und vielseitige Möglichkeit, sprachbezogene Aufgaben effizienter zu gestalten und mindestens partiell zu automatisieren.
